# Fitting the Fractional Polynomial Model to Non-Gaussian Longitudinal Data

**DOI:** 10.3389/fpsyg.2017.01431

**Published:** 2017-08-22

**Authors:** Ji Hoon Ryoo, Jeffrey D. Long, Greg W. Welch, Arthur Reynolds, Susan M. Swearer

**Affiliations:** ^1^Educational Leadership, Foundations, and Policy, University of Virginia Charlottesville, VA, United States; ^2^Department of Psychiatry, University of Iowa Iowa City, IA, United States; ^3^Buffett Early Childhood Institute, University of Nebraska Lincoln, NE, United States; ^4^Institute of Child Development, University of Minnesota Minneapolis, MN, United States; ^5^Department of Educational Psychology, University of Nebraska Lincoln, NE, United States

**Keywords:** fractional polynomial, generalized additive model, Non-Gaussian longitudinal data, Chicago longitudinal study, reading of the mind

## Abstract

As in cross sectional studies, longitudinal studies involve non-Gaussian data such as binomial, Poisson, gamma, and inverse-Gaussian distributions, and multivariate exponential families. A number of statistical tools have thus been developed to deal with non-Gaussian longitudinal data, including analytic techniques to estimate parameters in both fixed and random effects models. However, as yet growth modeling with non-Gaussian data is somewhat limited when considering the transformed expectation of the response via a linear predictor as a functional form of explanatory variables. In this study, we introduce a fractional polynomial model (FPM) that can be applied to model non-linear growth with non-Gaussian longitudinal data and demonstrate its use by fitting two empirical binary and count data models. The results clearly show the efficiency and flexibility of the FPM for such applications.

## Introduction

Just as in cross sectional studies, longitudinal studies frequently utilize non-Gaussian data that involve binomial, Poisson, Gamma, inverse-Gaussian distributions, and multivariate exponential families. Fitzmaurice and Molenberghs (2008) categorized models for non-Gaussian longitudinal data into three types based on the way they account for the correlation among the repeated measures and interpret the regression parameters: (1) marginal or population-average models, (2) generalized linear mixed models (GLMM), and (3) conditional and transition models. In the research reported here, we opted to use a GLMM as this emphasizes individual differences and takes into account random effects.

Given the prevalence of non-Gaussian longitudinal data, it is not surprising that a number of statistical tools have been developed for estimating parameters in both fixed and random effects models. Examples include Stiratelli et al. (1984) and Schall (1991), who proposed a penalized quasi-likelihood estimation; Pinheiro and Bates' (1995) Laplace approximation; and Breslow and Clayton (1993), who combined marginal quasi-likelihood estimation with a penalized quasi-likelihood estimation. Other approaches that have been proposed include Anderson and Aitkin's (1985) adaptive Gaussian quadrature; McCulloch's (1997) Monte Carlo EM algorithm; Kuk and Cheng's (1997) Monte Carlo Newton-Raphson algorithm; and Zeger and Karim's (1991) Monte Carlo integration via Gibbs sampling within the framework of the GLMM. However, in spite of the many estimating methods that have been suggested, growth modeling with non-Gaussian data remains somewhat limited when modeling the transformed expectation of the response when applying a linear predictor as a functional form for the explanatory variables.

As summarized by Fox (2016), the generalized linear model (GLM) consists of: (1) a random component specifying the conditional distribution of a response variable, *Y*_*i*_; (2) a linear predictor as a linear function of multiple regressors, η_*i*_ = α + β_1_*X*_*i*1_ + β_2_*X*_*i*2_ + … + β_*k*_*X*_*ik*_; and (3) a smooth and invertible linearizing link function, *g*(·), that transforms the expected value of the response variable to a linear predictor such as *g*(*E*(*Y*_*i*_)) = η_*i*_ = α + β_1_*X*_*i*1_ + β_2_*X*_*i*2_ + … + β_*k*_*X*_*ik*_. In the applications of GLM reported in the literature, the random component and the link function are generally well-organized, for example in the form of exponential families, as in Nelder and Wedderburn (1972). However, the functional form for the linear predictor has been largely restricted to a linear combination of explanatory variables, interactions, and polynomial regressors. Although the link function resolves the issue related to conditional distributions in the regression, the trends exhibited in the associations between the latent variable of a dependent variable and the explanatory variables within the linear predictor would be more various and complex. For example, the association could be an asymmetric curve that could not be resolved simply by interactions or polynomial regressors. In this paper, we introduce a more flexible functional form for the linear predictor, which is known as a fractional polynomial regressor (Royston and Altman, 1994; Long and Ryoo, 2010).

In the social and behavioral sciences, modeling individual differences in a change process is vital when compensating for a small sample and/or reducing sampling bias. Random effects models can be utilized to model these individual differences. A GLMM can be defined by adding random effects representing within-subject variations in the linear predictor (Laird and Ware, 1982):

(1) $$\begin{array}{l} \eta_{i}:=g\left(\mu_{i}\right)=\beta_{0}+\beta_{1}X_{i1}+\ldots +\beta_{p}X_{ip}+b_{0}+b_{1}Z_{i1}\\ \;+\;\ldots b_{q}Z_{iq}={\text{X}}_{i}\beta +{\text{Z}}_{i}b \end{array}$$

where μ_*i*_ is the expected value of the response variable, χ_*i*_ is a known design matrix linking β and η_*i*_ and *Z*_*i*_ is a known design matrix linking *b* and η_*i*_. In practice, *Z*_*i*_ is often constrained within the random intercept model, since non-Gaussian data provide relatively little information about individual heterogeneity beyond variability in the random intercept (Long et al., 2009). In the work reported here, *Z*_*i*_ is extended to random slope variability to account for non-Gaussian data, including both count data and binary data.

In longitudinal data, the time variable is treated as a covariate due to its key role in modeling change over time. For that reason, the first step in model selection involves a visual inspection of mean change over time (Pinheiro and Bates, 2000; Molenberghs and Verbeke, 2005). Though the result of the visual inspection is subjective and not necessarily representative, it does establish a starting point for the analyses. In this step, a generalized additive model (GAM; Hastie and Tibshirani, 1986; Berhane and Tibshirani, 1998) can offer useful support for the results obtained by a visual inspection of the data. As a non-parametric analytic tool, GAM is well-known and performs better than the local regression model (LOWESS; Cleveland, 1979; Cleveland and Devlin, 1988) when modeling the relationship between a response variable and each predictor.

To model the time transformation and articulate change over time, a conventional polynomial model (CPM) is typically utilized to identify the function with the best fit (Fitzmaurice et al., 2004, chap. 12). However, a CPM seldom offers the optimum solution and neither does it provide useful descriptive information, since the CPM is always symmetric around the local maxima and local minima and also requires an additional parameter for each fluctuation in the pattern of change. There are many alternatives to modeling asymmetric trends that provide more parsimonious models with the same quality of information on model fit (Sterba, 2014). Here, we focus on the use of fractional polynomial models (FPMs; Royston and Altman, 1994). Compared with CPMs, FPMs have received relatively little attention in the context of non-Gaussian longitudinal data in the social and behavioral sciences, even though FPMs are more flexible than CPMs and provide broader classes for model selection. The flexibility and parsimoniousness of FPMs in model selection for Gaussian longitudinal data have been discussed in Long and Ryoo (2010). This paper investigates model selection in the linear predictor for non-Gaussian longitudinal data, with an extension of the time transformation and random effects onto FPMs. This is also referred to as the generalized fractional polynomial mixed model (GFPMM).

## Methods

Two real world datasets were analyzed to demonstrate a unified framework for fitting GFPMM. The model selection procedure for GFPMM suggested by Ryoo (2011) in LMM was used to demonstrate the process involved in building the model by fitting the time transformation, static predictor, interactions, and random effects in Equation (1).

### Data sources

To demonstrate the parsimoniousness and flexibility in fitting non-Gaussian data using the GFPMM, two longitudinal datasets including binary and count responses were used. The first dataset consisted of a binary response over four time points, while the second dataset consisted of a count response over three time points. In the second dataset, two static predictors were also considered. In this exploratory data analysis, those two variables were tested to determine whether they were significant variables for the best fitting model.

### Chicago longitudinal study

As part of the Chicago Longitudinal Study (CLS, http://www.cehd.umn.edu/icd/cls/), data were collected from 1,539 Chicago public school students deemed to be at risk of educational and social difficulties due to economic disadvantage. Based on parents and teachers' surveys at ages 3, 8, 12, and 17, a risk index was computed by combining risk factors such as mother's age, mother's education level, single parenting status, number of children in the household, eligibility for free school lunches, mother's employment, and the poverty index in the local school area, a total of four data points for each child. The scale of the ordinal risk index from 0 to 8 was then further dichotomized as 0 for low risk (<4) and 1 for high risk (4 or higher). In this demonstration, the change in the proportion of high risk students was examined by applying GFPMM.

**Figure 1:**
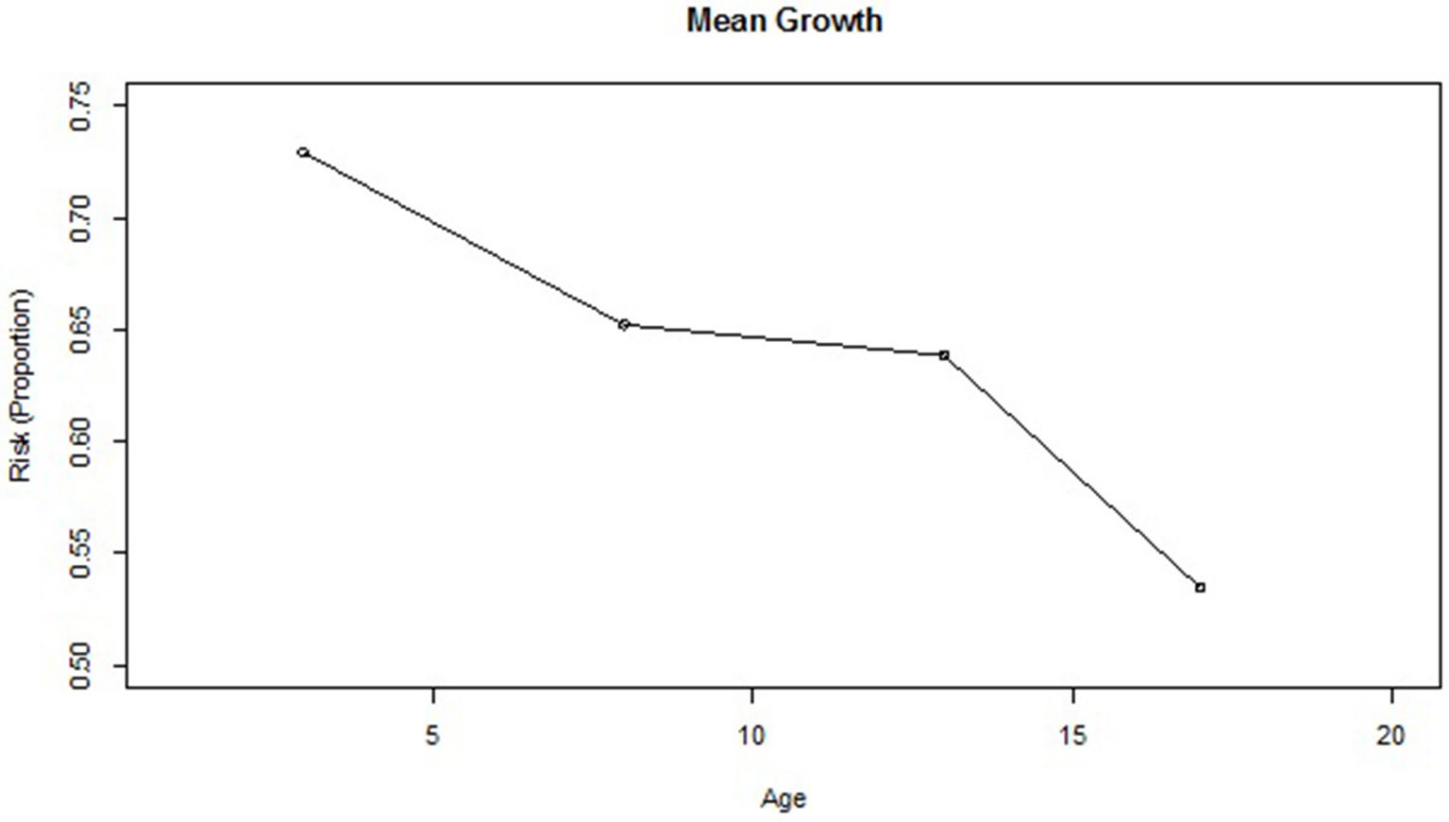
Mean change of risk index (Proportion) in the CLS dataset.

**Figure 2:**
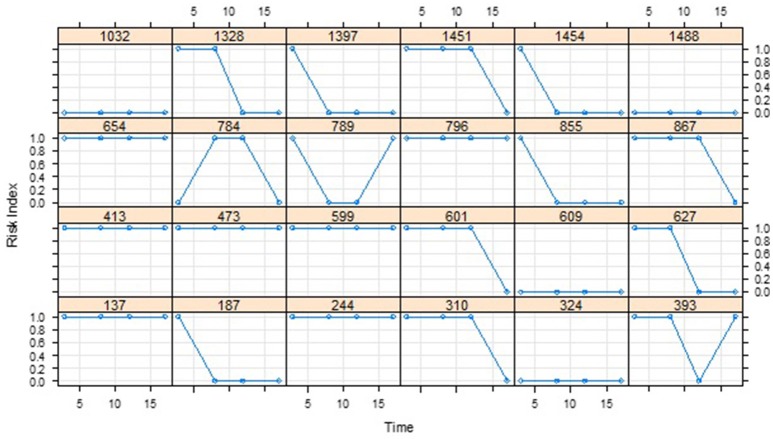
Individual curves for 24 participants randomly selected from the CLS dataset.

Based on the dichotomous classification risk index for each of the 1,539 students included in the dataset, 1,122 (72.9%), 1,004 (65.2%), 983 (63.9%), and 823 (53.5%) were deemed to be at-risk at ages 3, 8, 12, and 17 years, respectively. Plotting the mean proportion changes (Figure 1), the numbers of at-risk students clearly decrease over time in a non-linear manner, with the proportion at age 17 dropping significantly below that at age 12. To further investigate the trend, the non-linear relationship between the binary response variable and the continuous time variable was modeled by applying GFPMM and GAM. To visualize the individual differences in terms of the change of at-risk status, we also plotted separate curves for 24 individuals randomly selected from the 1,539 participants (Figure 2). Within these small randomly selected datasets, we observed nine difference patterns in terms of their at-risk status. To articulate both the overall change and individual change patterns, we utilized GAM analysis as a diagnostic tool to capture these patterns.

### Reading of the mind in the eyes test-revised

The Reading of the Mind in the Eyes Test-Revised (RMET-R; Baron-Cohen et al., 2001) is a 28-item measure designed to assess children's ability to make social judgments based on stimuli such as the eye region of another person's face. This test involves 28 photographs of the facial eye region, each accompanied by four words describing various mental states. In the present study, 212 participants were asked to select the word that best described the mental state exhibited in the accompanying image, with a point awarded for each correct answer. The points were then summed to compute an overall score representing the participant's response at each time point, calculated as 28 minus the total score. Lower scores thus indicate higher “Theory of Mind.” Data were collected at three different time points spaced at ~6 month intervals. Two demographic variables, gender (1 for girl and 2 for boy) and first language (1 for English and 2 for Others), are included in this demonstration.

**Figure 3:**
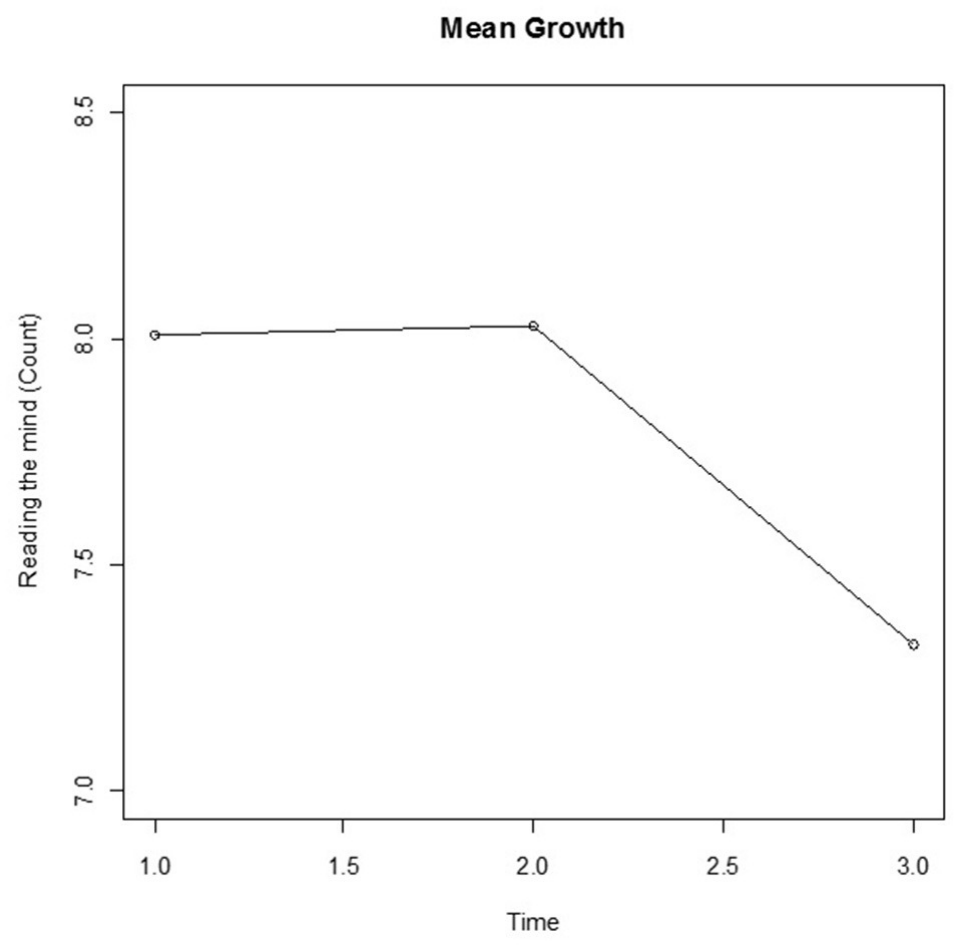
Mean change of total score (Count) for the RMET-R dataset.

**Figure 4:**
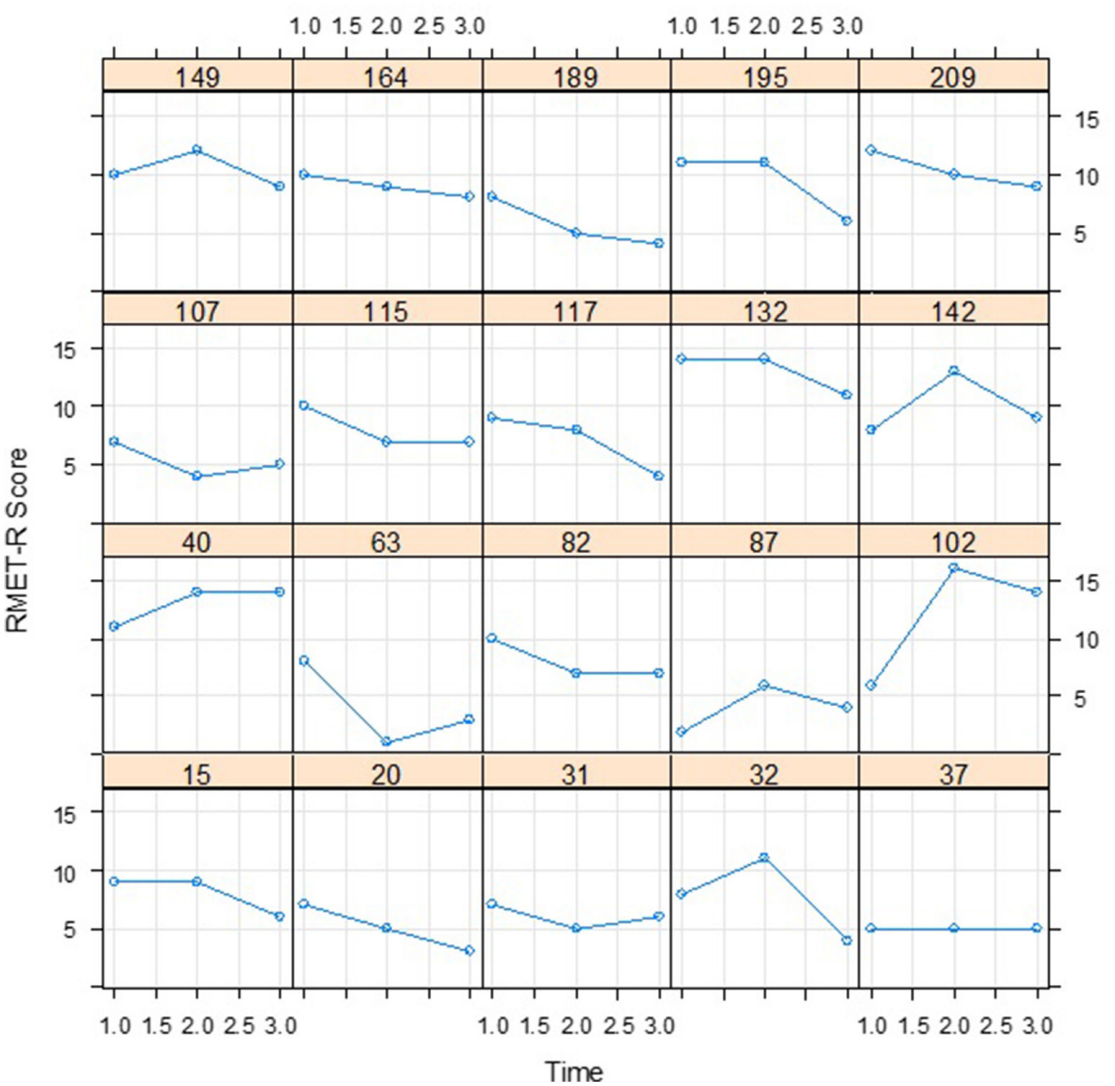
Individual curves for 20 participants randomly selected from the RMET-R dataset.

The means for the RMET-R test results are plotted in Figure 3. At first glance, the sharp drop between Time 2 and Time 3 would seem to indicate that a non-linear model would be more appropriate for modeling the change than a linear model. However, as the data presented in Figure 4 suggests, this may not in fact be the case; the wide variations in the individual curves for 20 randomly selected participants may indicate non-linearity and decreases over time. To articulate both the overall and individual change patterns, we therefore opted to utilize GAM analysis as a diagnostic tool in order to capture both patterns.

### Generalized additive model (GAM)

To aid in the visual inspection of the data, GAM incorporating non-parametric regression and smoothing was utilized. GAM was initially used to model the change at the group level by taking individual changes into account. Unlike GLM, the linear predictor for GAM uses smooth functions rather than linear combination of predictors as follows:

(2) $$\eta =s_{0}+\sum_{i=1}^{p}s_{i}(X_{i})$$

where *s*_1_(·), … , *s*_*p*_(·) are smooth functions.

Applying back-fitting and local scoring algorithms and generalized cross validation (GCV; Wahba, 1990), GAM estimates the smooth functions and consequently provides a means of examining possible non-linear change between the response variable and each predictor variable.

### Generalized fractional polynomial mixed model (GFPMM)

**Figure 5:**
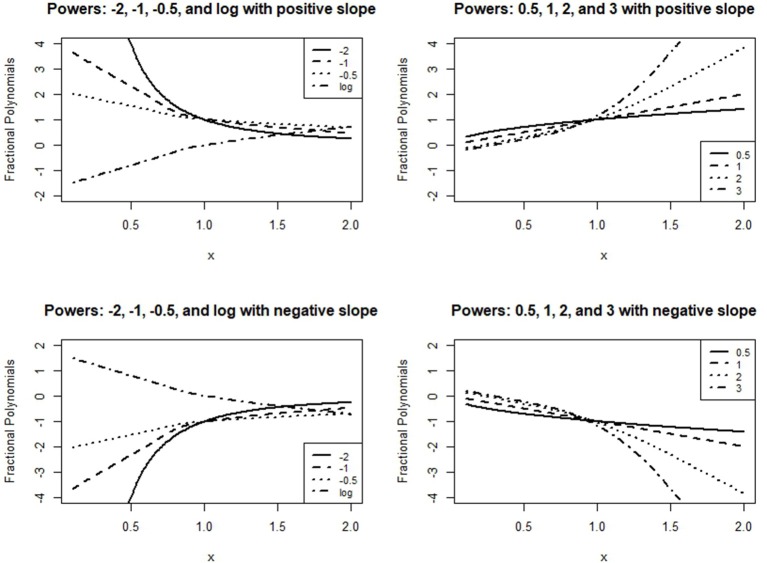
First order fractional polynomials.

The primary statistical model utilized in this study was GFPMM, which can be written in terms of the linear predictor, η_*ij*_, using the following the individual level form:

(3) $$\begin{array}{l} \eta_{ij}=\beta_{0}+\sum_{b=1}^{p}\beta_{b}f_{b}(t_{ij})+\sum_{e=1}^{l}\gamma_{e}X_{ej}+b_{i0}+\sum_{g=1}^{q}b_{ig}f_{g}(t_{ij})\\ \;=\;{\text{X}}_{i}\beta +{\text{Z}}_{i}b \end{array}$$

where *p* and *q* indicate the orders of the FP terms for the time variable for the fixed effects and the random effects, respectively, and *l* indicates the number of static predictors. The indices *i* and *j* denote the *i*th time and *j*th person, respectively. An FP function, *f*, is defined as:

(4) $$f_{h}(y_{ij})=\{\begin{array}{cc} y_{ij}^{\left(m_{h}\right)}, & \text{if }m_{h}\neq m_{h-1},\\ f_{h-1}(y_{ij})\cdot \log(y_{ij}), & \text{if }m_{h}=m_{h-1}, \end{array}$$

where *m*_1_ ≤ *m*_2_ ≤ … ≤ *m*_*p*_. The parentheses on the exponent signify Box and Tidwell's (1962) transformation:

(5) $$y_{ij}^{\left(m_{h}\right)}=\{\begin{array}{cc} y_{ij}^{m_{h}}, & \text{if }m_{h}\neq 0,\\ \log(y_{ij}), & \text{if }m_{h}=0, \end{array}$$

with the constraint *y*_*ij*_ > 0, so that all transformations are defined. The power terms (exponents), *m*_*h*_, are taken from the set of values suggested by Royston and Altman (1994) for general curve fitting. The set is:

(6) M={−2,−1,−0.5, 0, 0.5, 1, 2, max(3,m)}

where *m* is the order of the FP. The values in *M* offer a wide variety of curve shapes and constitute transformations that are familiar to applied researchers. Figure 5 presents the shapes of the first order FPs, demonstrating this approach's flexibility in terms of fitting a fixed model.

In the equation for the linear predictor, η_*ij*_, the first three terms refer to the fixed effects and the last two to the random effects. Applied researchers may also be interested in the interactions between time variables and additional explanatory variables. This term can be added by multiplying the 2nd and 3rd terms in the equation for the linear predictor, η_*ij*_. If no between-person variation is expected, the random effects terms can be dropped.

### Maximum likelihood estimation for GFPMM

Within the format of a GLMM, GFPMM can also be estimated using one of three common estimation methods: penalized quasi-likelihood (PQL; Schall, 1991; Breslow and Clayton, 1993), integral approximations such as the Laplace approximation (Pinheiro and Bates, 1995), or Gauss-Hermite quadrature (McCulloch, 1997). In the current study, we used the Laplace approximation as we consider it to obtain more accurate results than PQL and to avoid the additional computational burden imposed by Gauss-Hermite quadrature.

The GFPMM can be written in matrix format as follows:

(7) η=X·β+Z·b and g(μ)=g[E(y|b)]=η

where *b*~N (0,Ψ). The mean and variance conditioned random effects can also be written as *E*(*y*|*b*) = μ and *V*(*y*|*b*) = φ · ν(μ), respectively, where φ is a dispersion parameter and ν(μ) is a variance function that depends on the distribution family. Based on the multivariate normality assumption for ν(μ), we can estimate the fixed effects β, along with the dispersion parameter φ, by maximizing the marginal distribution:

(8) $$p(y|\beta ,\varphi )=\int_{b}p(y|\beta ,\varphi ,b)p(b|\Psi )db$$

We can now re-estimate the variance function, ν(μ), using a pseudo-variable, y^*^, that is predicted by the linear predictor. Estimation continues in this manner until convergence is achieved. The R lme4 package (Bates et al., 2015) was used to estimate the parameters in GFPMM.

### Model comparison

After diagnosing the trends in the change patterns using mean changes and the GAM results, we applied the generalized conventional polynomial mixed model (GCPMM) to both datasets. We considered conventional up to quadratic polynomials for the CLS data and a linear model for the RMET-R data with up to random slope models due to their respective four and three time points. The best fit was obtained for the GFPMM with random slope models. We then compared the best fitting GCPMM and GFPMM to identify the model with the best fit. When comparing models, we utilized a generalized likelihood ratio test (GLRT; Cox and Hinkley, 1974; Wood, 2006) with a significance level of 0.05, and delta (Δ) methods (Burnham and Anderson, 2004) based on AICc (Hurvich and Tsai, 1989) and BIC (Schwarz, 1978), applying the criteria that models with Δ_*i*_: = Δ*AICc*_*i*_ = *AICc*_*i*_ − min(*AICc*) ≤ 2 have substantial support, models with 4 ≤ Δ_*i*_ ≤ 7 have considerably less support, and models with Δ_*i*_ > 10 have essentially no support. This last delta criterion has also been applied to BIC in similar applications in the Bayesian literature (Raftery, 1996).

## Results

### Chicago longitudinal study dataset

As shown in Figure 1, the proportion of high risk students drops from age 3 to age 8, levels off to some extent from age 8 to age 12, and then drops sharply from age 12 to age 17. This is somewhat inconsistent with the results obtained from the GAM analyses, which generates the linear model shown. The GAM curve follows a slightly different pattern from the mean change shown in Figure 1, indicating a decreasing pattern and faster drop for the oldest students. Interestingly, the GAM analysis yields an effective degree of freedom of one, meaning that the suggested value for the exponent of the time variable is linear. Based on both these findings, the quadratic polynomial model was considered to provide the best fit for the time transformation.

Analyses using the polynomial methods begin by implementing GCPMM. In the class of conventional polynomials with four time points, the suggested model would be considered to be either linear or quadratic polynomial; a cubic model would be a saturated model for fixed effects. For random effects, the models up to the random slope term were considered in a well-formulated model. The well-formulated model includes lower order terms when higher order terms are significant, regardless of the significance of the lower order terms (Morrell et al., 1997). Table 2 displays the results of the competing models utilizing GCPMM.

The results shown in Table 1 suggest that the best fit is obtained for the quadratic model with linear random effects, described in equation form as follows:

(9) $$\begin{array}{l} \log\left\{\frac{\mathrm{Pr}(Y_{ij}=1|b_{0i},b_{1i})}{\mathrm{Pr}(Y_{ij}=0|b_{0i},b_{1i})}\right\}=\eta_{ij}=\beta_{0}+\beta_{1}\cdot time_{ij}+\beta_{2}\cdot time_{ij}^{2}\\ \;+\;b_{0i}+b_{1i}\cdot time_{ij} \end{array}$$

The resulting GCPMM does not adequately describe the drops observed in the results for the quadratic model, however, since conventional polynomials are symmetric around vertexes. On the other hand, the shapes of fractional polynomials vary more widely than conventional polynomials, as can be seen in Figure 5. In addition, FPMs can be fit with a relatively small number of parameters; although the GCPMM with the best fit (Equation 9) includes six parameters, the GFPMM with the best fit (Equation 10) includes only five. The results of the model selection for the random intercept and intercept FPM are summarized in Table 2. The results suggest the FPM with power 3 provides the best fit in terms of the two delta methods, BIC and AICc; delta values for the GFPMM with power −1 are >10. Thus, the model with the best fit for GFPMM combines a cubic polynomial term with random slope effects and can be written in equation form as follows:

(10) $$\begin{array}{l} \log\left\{\frac{\mathrm{Pr}(Y_{ij}=1|b_{0i},b_{1i})}{\mathrm{Pr}(Y_{ij}=0|b_{0i},b_{1i})}\right\}=\eta_{ij}=\beta_{0}+\beta_{1}\cdot time_{ij}^{3}+b_{0i}\\ \;+\;b_{1i}\cdot time_{ij}^{3} \end{array}$$

**Table 1:** Results of model selection within generalized conventional polynomial mixed models (GCPMMs) for the CLS dataset.

**Models**	**Number of parameters**	**BIC**	**AICC**	**GLRT**	**Δ(BIC)**	**Δ(AICC)**
				**Chisq (*p*-value)**		
gcpm.10^a^	3	6,179	6,159		52.46	69.03
gcpm.20^b^	4	6,186	6,159	1.24 (0.27)	59.94	69.79
gcpm.11^c^	5	6,126	6,093	68.67 (0.00)	0.00	3.13
gcpm.21^d^	6	6,130	6,090	5.13 (0.02)	3.59	0.00

a Random intercept linear model;

b Random intercept quadratic model;

c Linear model with random slope;

d *Quadratic model with random slope*.

**Table 2:** Results of model selection in 1st order generalized fractional polynomial mixed models (GFPMMs) with random intercept and slope for the CLS dataset.

	**Power**	**BIC**	**AICC**	**Δ(BIC)**	**Δ(AICC)**
Random intercept GFPMM (df = 3)	−2	6,149	6,129	151.15	164.60
	−1	6,121	6,101	123.88	137.33
	−0.5	6,103	6,083	105.20	118.64
	0	6,084	6,064	86.43	99.87
	0.5	6,069	6,049	71.85	85.30
	1	6,062	6,042	64.46	77.91
	2	6,066	6,046	68.46	81.91
	3	6,079	6,059	81.92	95.36
Random slope GFPMM (df = 5)	−2	6,278	6,244	280.02	280.02
	−1	6,031	5,997	33.41	33.41
	−0.5	6,073	6,039	75.07	75.07
	0	6,098	6,065	100.61	100.61
	0.5	6,113	6,080	115.84	115.84
	1	6,126	6,093	128.73	128.73
	2	6,147	6,113	149.25	149.25
	3	5,998	5,964	0.00	0.00

**Figure 6:**
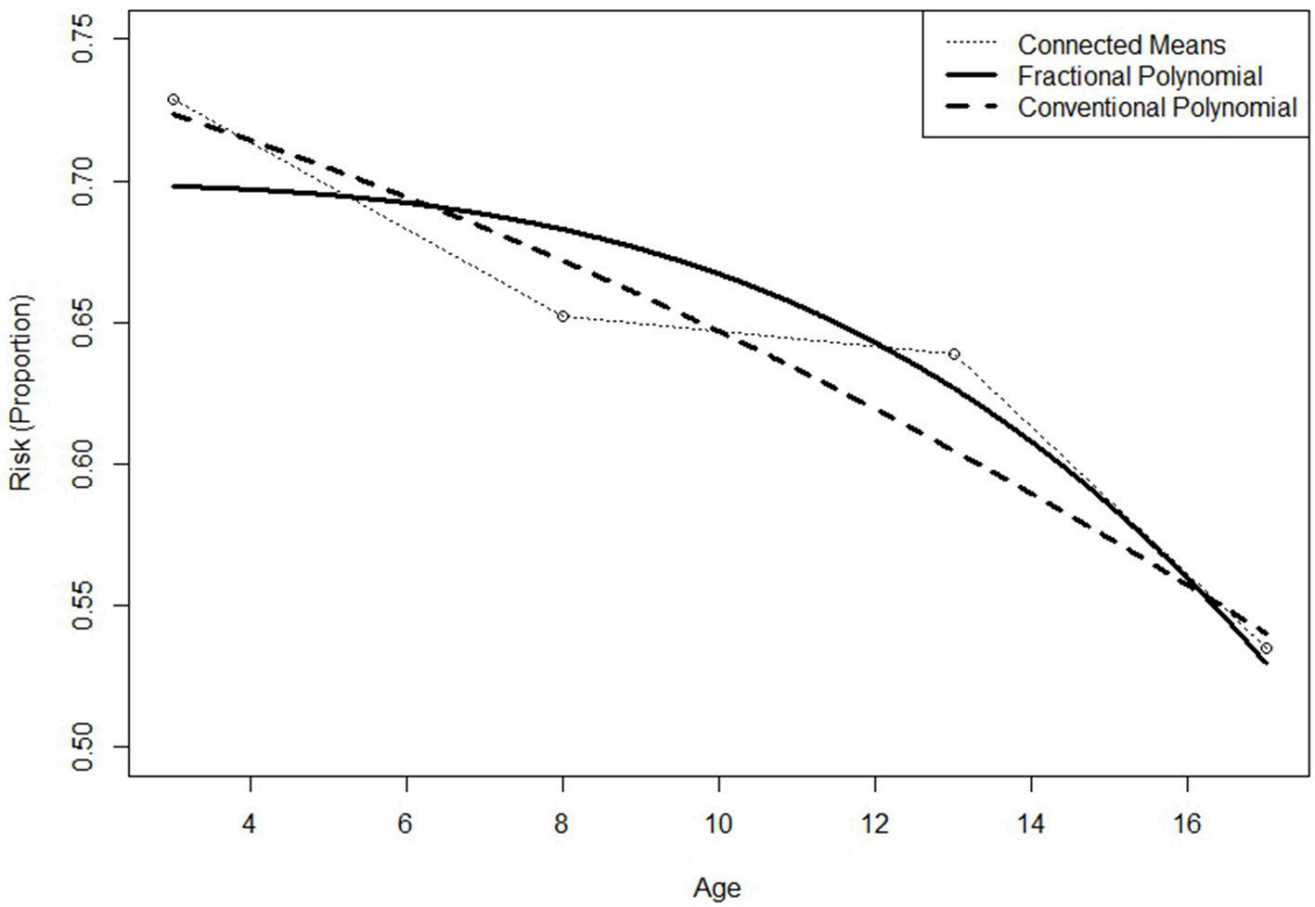
Predicted curves for the 2nd order generalized conventional polynomial (GCPMM) and 1st order generalized fractional polynomial mixed model (GFPMM) with mean changes for the CLS dataset.

When comparing the results of the best fitting models for GCPMM and GFPMM, GFPMM performs better than the best GCPMM, as indicated by the Δ(BIC) and Δ(AICC) values of 132.3 and 125.6, respectively, in Table 3. This result is also supported by Figure 6, where the GFPMM with the best fit shows a stable decrease between ages 3 and 12, at which point there is a sudden drop.

**Table 3:** Results of model comparison between the best fitted GCPMM and GFPMM for the CLS dataset.

**Models**	**Number of parameters**	**BIC**	**AICC**	**GLRT**	**Δ(BIC)**	**Δ(AICC)**
				**Chisq (*p*-value)**		
GFPMM^a^	5	5998	5964		0.0	0.0
GCPMM^b^	6	6130	6090	0.000 (1.000)	132.3	125.6

a 1st order fractional polynomial model with power 3 and random slope;

b *Quadratic polynomial model with random slope*.

### RMET-R

The RMET-R data consists of only three time points. Figure 3 displays the mean changes, with a relatively stable value from Time 1 to Time 2 followed by a decrease from Time 2 to Time 3, which is similar to the results of the GAM analysis. However, the 20 randomly selected individual curves from among the 212 participants plotted in Figure 4 fail to exhibit any coherent pattern but instead clearly show the between-subject variability. This random effect was thus taken into account for the between-subject variability. In the GCPMM class, the random effects can be either random intercept or random slope.

Both the GLRT and the delta method indicate that the random slope model performs significantly better than the random intercept model (Table 4). Next, variable selections were conducted via the GLRT and delta methods to select the variable that most affects the model. The result of both methods indicated that language is not significant but gender is. Interestingly, unlike the main effect of the gender variable, the interaction effect between the gender and time variables was not significant. The results for the gender variable are summarized in Table 4. The GCPMM with the best fit can be written as follows:

(11) $$\begin{array}{l} \log\left\{E(Y_{ij}|b_{i})\right\}=\eta_{ij}=\beta_{0}+\beta_{1}\cdot time_{ij}+\beta_{2}\cdot gender_{i}+b_{0i}\\ \;+\;b_{1i}\cdot time_{ij} \end{array}$$

**Table 4:** Results of model selection within GCPMM for the “Reading the Mind” dataset (*N* = 212).

**Models**	**Number of parameters**	**BIC**	**AICC**	**GLRT**	**Δ(BIC)**	**Δ(AICC)**
				**Chisq (*p*-value)**		
gcpm.10^a^	3	885.846	872.519		129.960	142.259
gcpm.11^b^	5	755.887	733.706	142.870 (0.000)	0.000	3.446
gcpm.11g^c^	6	756.857	730.260	5.485 (0.019)	0.971	0.000
gcpm.11gi^d^	7	762.065	731.056	1.248 (0.264)	6.178	0.797

a *Random intercept linear model*.

b *Linear model with random slope*.

c *Linear model with random slope and gender*.

d *Linear model with random slope, gender, and interaction between gender and time*.

Next, the class of model was extended to GFPMM. As can be seen in Figure 3, the mean remains roughly constant between Times 1 and 2 but then drops off sharply. The model with the best fit thus needs to describe this behavior over time. Among the random intercept models tested, this would appear to be the GFPMM with power 3 based on the results presented in Table 5. However, the GFPMMs with powers 1, 2, and 3 all have substantial support based on the criteria suggested by Burnham and Anderson (2004) so instead of selecting a single model, these three GFPMMs were all considered candidates for the best fit.

**Table 5:** Model selection in the 1st order generalized fractional polynomial mixed models (GFPMMs) with random intercept and slope for the “Reading the Mind” dataset.

	**Power**	**BIC**	**AICC**	**Δ(BIC)**	**Δ(AICC)**
Random intercept GFPMM (df = 3)	−2	889.069	875.742	153.200	162.054
	−1	888.242	874.914	152.373	161.226
	−0.5	887.703	874.375	151.833	160.687
	0	887.100	873.772	151.230	160.084
	0.5	886.466	873.139	150.597	159.451
	1	885.846	872.519	149.977	158.831
	2	884.803	871.475	148.933	157.787
	3	884.126	870.799	148.257	157.111
Random slope GFPMM (df = 5)	−2	737.510	715.329	1.641	1.641
	−1	736.963	714.782	1.094	1.094
	−0.5	736.658	714.477	0.789	0.789
	0	736.365	714.185	0.496	0.497
	0.5	737.776	715.595	1.907	1.907
	1	755.887	733.706	20.018	20.018
	2	735.869	713.688	0.000	0.000
	3	736.076	713.895	0.207	0.207

As discussed in the Introduction Section, the random effects were extended to the random slope. The results, summarized in Table 5, suggests that all the GFPMMs except the GFPMM with power 1 have substantial support. Regardless of the structure of the random effects, the GFPMMs with powers 2 and 3 have substantial support. Since both these GFPMMs performed better in the further model comparison, they are jointly considered the best fitted models.

**Figure 7:**
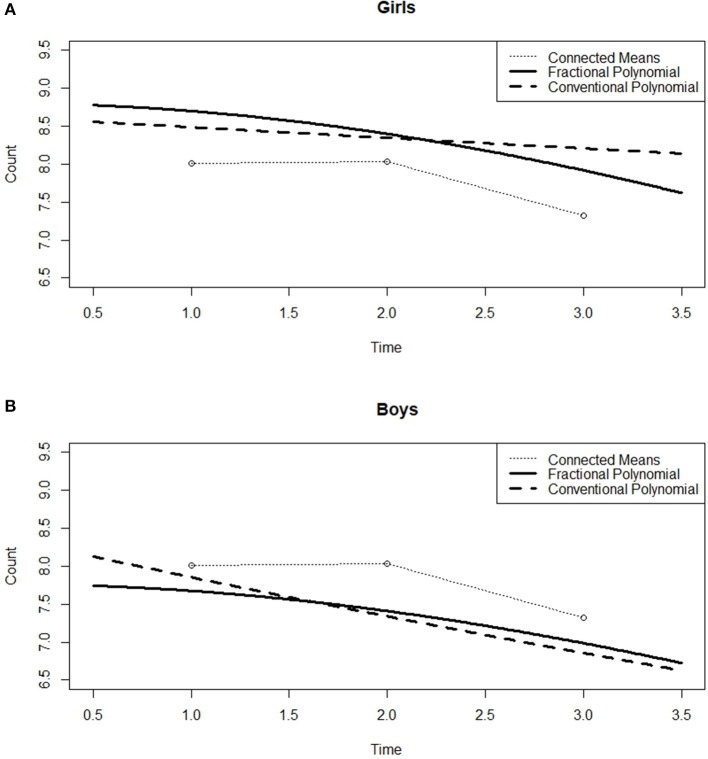
Predicted curves for GCPMM and GFPMM with mean changes across gender for the RMET-R dataset. **(A)** Girls, **(B)** Boys.

We further investigated the effect of gender by fitting all eight GFPMMs with random slopes. In all the 1st order GFPMs, the gender is significant (all of *p* ≤ 0.032); although there is no interaction effect with the time variable (all of *p* ≥ 0.110) at a significance level of 0.05. As a result, the gender variable was added to the model as a main effect. This result can be confirmed by examining Figure 7. As the figure shows, the mean changes for boys and girls differ slightly. Comparing these mean changes with those predicted by the GFPMM with power 2 and the linear GCPMM, the mean for the girls is lower than that for the boys, which indicates that as a group, the girls have a higher “Theory of Mind” than the boys.

After the gender variable was added into the 1st order GFPMMs, all the 1st order GFPMMs were compared and the results are summarized in Table 6. Although five of the GFPMMs have substantial support, the GFPMM with power 2 has the smallest BIC and AIC_*C*_. The model equation can thus be written as follows:

(12) $$\begin{array}{l} \log\left\{E(Y_{ij}|b_{i})\right\}=\eta_{ij}=\beta_{0}+\beta_{1}\cdot time_{ij}^{2}+\beta_{2}\cdot gender_{i}+b_{0i}\\ \;+\;b_{1i}\cdot time_{ij}^{2} \end{array}$$

**Table 6:** Results of model selection in the 1st order generalized fractional polynomial mixed model (GFPMM) with random slope and gender for the “Reading the Mind” dataset.

**Power**	**BIC**	**AICC**	**Δ(BIC)**	**Δ(AICC)**
−2	739.359	712.761	2.052	2.051
−1	738.765	712.167	1.458	1.457
−0.5	738.423	711.825	1.116	1.115
0	738.081	711.483	0.774	0.773
0.5	739.555	712.957	2.248	2.247
1	756.857	730.260	19.550	19.550
2	737.307	710.710	0.000	0.000
3	737.381	710.783	0.074	0.073

Within the various conditions tested for the FPMs, the models with the best fit are the GFPMM with power 2 in terms of BIC and the GFPMM with power 2 and the gender variable in terms of AICc. Both GFPMMs have random slope as their random effect and both are substantially supported compared with the best fitted models shown in Table 7. However, GLRT indicates that the GFPMM with power 2 and the gender variable performs significantly better than the GFPMM with power 2 alone.

**Table 7:** Results of model comparison between the best fitted GCPMM and GFPMM in the “Reading the Mind” dataset.

**Models**	**Number of parameters**	**BIC**	**AICC**	**GLRT**	**Δ(BIC)**	**Δ(AICC)**
				**Chisq (*p*-value)**		
GFPMM^a^	5	735.869	713.688		0.000	2.979
GFPMM^b^	6	737.307	710.710	5.017 (0.025)	1.438	0.000
GCPMM^c^	6	756.857	730.260	0.000 (1.000)	20.988	19.550

a 1st order fractional polynomial model with power 2 and random slope;

b 1st order fractional polynomial model with power 2, random slope, and gender;

c *Linear model with random slope and gender*.

## Discussion

In this study, the efficiency and parsimoniousness of GFPMM have been investigated for non-Gaussian longitudinal data, specifically in the context of binary and count responses. In line with the flexibility of shapes in FPMs, the GFPMMs with the best fit performed better than GCPMMs for both types of data according to the GLRT and delta methods used to assess the results of two empirical studies. In addition, GFPMMs are more parsimonious than GCPMMs. As shown in the analyses above, GFPMMs can be utilized to achieve more parsimonious (i.e., requiring fewer parameters), better fitting models than GCPMMs.

The results of the GAM analyses provide fairly consistent models for the GFPMMs with the best fits, which shows that the non-parametric modeling is well-incorporated with the parametric modeling conducted via standard model comparison tools, namely the GLRT and delta methods. Such compatibility is not achievable with the comparable GCPMMs due to the limited choice of shapes available. Thus, for the case of fitting non-linear trends in non-Gaussian longitudinal data, we recommend that applied researchers utilize GFPMM with GAM and using the GLRT and delta methods for their modeling. In this paper, we mainly focused on exploratory analysis. However, it is not limited to exploratory analysis but the procedure can also be used for any confirmatory analysis.

We have also demonstrated the use of GFPMM in the context of non-linear patterns of change. GFPMM offers a flexible and efficient means of modeling non-linear change in continuous, binary, and count data within the linear predictor. As our analyses of the CLS and RMET-R datasets showed, GFPMM can provide a more parsimonious solution than the traditional approach based on GCPMM.

The utility of this approach does require further investigation in the context of a multitude of different data situations where non-linear change is present. In general, the main difficulty in fitting polynomial models lies in the interpretation of the results. That is, what does the model tell us about the substantive nature of the data? Answering this question lies at the heart of every investigation undertaken by applied researchers. In the context of the types of data illustrated above, a better model fit was achieved by using GFPMM rather than GCPMM. Of course, the need to estimate fewer parameters inevitably eases the interpretation of the model and in the cases illustrated above GFPMM provided a better model fit with fewer parameters.

Royston and Altman (1994) noted that a GFPMM with order >3 is rarely fitted due to its inherent complexity, which does not actually mean that their flexibility is limited but rather that GFPMMs up to order two provide a wide variety of shapes that are generally sufficient to model non-linear trends in longitudinal data. The shapes of a 2nd order GFPMM can be found in Long and Ryoo (2010). If there are many time points, then dynamic changes would be expected in practice. In the case, higher order GFPMMs can also be applicable. Such flexible and parsimonious properties in the GFPMM suggest applied researchers to consider it when asymmetric growth pattern is observed or expected. Although a number of time points will not be associated with using the GFPMM, three to six time points often favor the GFPMM than the GCPMM (Long and Ryoo, 2010; Ryoo et al., 2017). Future studies will examine the efficiency via a simulation study. By considering the random effects introduced to take in account the between-person variability, GFPMM incorporated with GAM provides an advanced statistical model that takes care of these individual differences. In the social and behavioral sciences, the articulation of individual difference is of paramount importance. Thus, fitting GFPMM to non-Gaussian longitudinal data will enable applied researchers in these areas to articulate individual differences in model selection.

## Ethics statement

All procedures performed in studies involving human participants were in accordance with the ethical standards of the institutional and/or national research committee and with the 1964 Helsinki declaration and its later amendments or comparable ethical standards.

## Author contributions

JR designed this study, performed the statistical analysis, and drafted the manuscript. JL and GW reviewed analysis, and drafted the manuscript; AR collected data and drafted the manuscript; SS collected data and drafted the manuscript. All authors read and approved the final manuscript.

### Conflict of interest statement

The authors declare that the research was conducted in the absence of any commercial or financial relationships that could be construed as a potential conflict of interest.
